# Plasma orexin-A and ghrelin levels in patients with chronic obstructive pulmonary disease: Interaction with nutritional status and body composition

**DOI:** 10.3892/etm.2014.1611

**Published:** 2014-03-10

**Authors:** GAMZE AKBULUT, MAKBULE GEZMEN-KARADAĞ, YASEMİN ERTAŞ, BANUGÜL BARUT UYAR, EMİNE YASSIBAŞ, DUYGU TÜRKÖZÜ, FERİDE ÇELEBİ, ÖZGE TUĞÇE PAŞAOĞLU, ONUR TOKA, HİLAL YILDIRAN, NEVİN ŞANLIER, NURDAN KÖKTÜRK

**Affiliations:** 1 Department of Nutrition and Dietetics, Faculty of Health Sciences, Gazi University, Ankara 06500, Turkey; 2 Department of Statistics, Hacettepe University, Ankara 06800, Turkey; 3 Department of Pulmonary Diseases, Faculty of Medicine, Gazi University, Ankara 06560, Turkey

**Keywords:** orexin-A, ghrelin, chronic obstructive pulmonary disease, diet, anthropometric measurements

## Abstract

Orexin-A and ghrelin are two important polypeptides that stimulate food intake, however, there is a lack of sufficient information concerning their plasma levels in patients with chronic obstructive pulmonary disease (COPD). The aim of the present study was to investigate the association between plasma orexin-A and ghrelin levels with food consumption and body composition in patients with stable phase COPD. In total, 40 patients (age, 44–80 years; male, 31; female 9) who were in the stable phase of COPD were included in the study. Blood samples for plasma orexin-A and ghrelin analysis were collected after 8–12 h of fasting; certain anthropometric measurements were obtained and a 24-h dietary recall was recorded. The mean plasma orexin-A levels in the male and female patients were 1.3±0.37 and 1.4±0.13 ng/ml, respectively, while the mean plasma ghrelin levels were 25.9±7.31 and 27.3±8.54 ng/ml, respectively. No significant correlation was observed between the body mass index and plasma orexin-A and ghrelin levels or between the plasma ghrelin levels and dietary nutrient intake (P>0.05). The plasma orexin-A levels were demonstrated to be higher in patients with a higher dietary total fibre intake (r=0.303, P=0.022). A similar correlation was observed between plasma orexin-A levels and dietary intake of soluble (r=0.033, P=0.029) and insoluble (r=0.335, P=0.024) fibre, as well as between the daily consumption of calcium and the levels of plasma orexin-A (r=0.065, P=0.046). Therefore, the results of the present study indicated that a positive correlation existed between dietary nutrient intake and plasma orexin-A levels in patients with COPD.

## Introduction

Chronic obstructive pulmonary disease (COPD) has a high incidence worldwide and a high mortality rate. According to data from the World Health Organization (WHO), it is predicted that COPD, which was the sixth most common cause of mortality in 1990, is likely to the third most common cause by 2020 (1,2).

Malnutrition is frequently reported in COPD patients and is an indicator of poor prognosis (3–8). Being underweight is associated with a high mortality rate in patients with COPD (7), which may be explained by the weakening of the respiratory and skeletal muscles (9).

Specific hypothalamic neuropeptides, including orexin, are generally affected by nutritional status and dietary food intake (10,11). Ghrelin is also an important polypeptide that stimulates food intake (12,13). Orexins play an important role in various physiological events, including the stress response and the sleep/wake cycle (14). In a previous study, orexin-A levels in patients with COPD were reported to be lower than those in the control group. Furthermore, the levels of orexin-A were found to be lower in underweight COPD patients when compared with those in normal weight patients with COPD (8). Ghrelin stimulates food intake, increases adiposity by decreasing lipid oxidation and maintains the energy balance (12,13,15). Itoh *et al* (16) indicated that plasma ghrelin levels were higher in underweight patients with COPD than in normal weight patients with COPD.

There are a limited number of studies that evaluate the association between plasma orexin-A and ghrelin levels with body composition and dietary intake in patients with COPD. To the best of our knowledge, there are no similar studies concerning patients with COPD in Turkey. For this reason, the present study aimed to investigate a possible correlation between plasma orexin-A and ghrelin levels with body composition and food consumption.

## Materials and methods

### Subjects

A total of 40 patients with stable phase COPD (44–80 years old) who were admitted to the Department of Pulmonary Diseases, Faculty of Medicine, Gazi University (Ankara, Turkey) between July 2012 and July 2013 volunteered for this study. The study was conducted in accordance with the regulations of Gazi University Clinical Research Ethics Committee and each participant signed a voluntary participation form. Due to financial problems, we did not use control patients in this research.

### Experimental design

Following a fasting period of 8–12 h, 7-ml blood samples were extracted from the patients and into tubes containing EDTA (Phoenix Pharmaceuticals, Inc., Belmont, CA, USA). A protease inhibitor, aprotinin, was added to the tubes (100 μl/ml blood), as recommended by the manufacturer of the ELISA kits (Phoenix Pharmaceuticals, Inc.). The samples were centrifuged at 1,600 × g at 4°C for 15 min. The plasma samples were separated and maintained at −32°C until required for analysis (17,18). The samples were analysed in duplicates.

Orexin-A and ghrelin levels in the plasma samples were analysed via an ELISA method, an immunoassay that determines antigen-antibody interactions, using ELISA kits purchased from Phoenix Pharmaceuticals, Inc. (17,18). According to the manufacturer’s instructions, the minimum detection limits of orexin-A and ghrelin were 0.2 and 0.13 ng/ml, respectively.

Data were collected from the patients during a face-to-face interview with qualified dieticians. In the interview, a questionnaire was used to determine the general features (age, gender, educational status and occupation), smoking habits, alcohol use and diet habits of the patients. Food consumption was measured with a 24-h dietary recall. During the interview, food models and photos of common Turkish foods of various portion sizes, as well as household cups and measures, were used to assess the type and amount of food and beverage consumed during the previous day (19). The average daily energy and macro- and micronutrient intakes were calculated using the computer software Nutrition Information Systems (BeBiS, Version 7.0, Pasific Company, Stuttgart, Germany). These values were compared with the recommended daily allowance values to determine the energy and nutrient requirement meeting status and the requirement meeting percentages were calculated (20). The dietary guidelines for Turkey were used for the assessment of specific nutrients (21).

All anthropometric measurements were obtained by trained dieticians, as described in a previous study (11). Body composition parameters, including weight, total body water, fat mass, lean mass and fat percentages of the patients, were measured using an InBody720 Bioelectrical Impedance Analyzer (Biospace, Seoul, Korea). The body mass index (BMI) was calculated as weight/(height)^2^ and BMI classifications were determined according to the WHO standards (22).

### Statistical analysis

Data analysis was performed using SPSS software, version 16.0 (SPSS, Inc., Chicago, IL, USA). As the data did not exhibit normal distribution, the median and interquartile range values were used to conduct statistical analyses of the daily dietary energy and nutrient intakes and body compositions, however, the mean and SD values are also presented. The Mann-Whitney U test was used for statistical comparison of the genders. Spearman’s correlation test was used to analyse correlations between the parameters, including the correlation between energy and nutrient intake and body composition with orexin-A and ghrelin levels; regression analysis was performed for the associated parameters. The statistical significance level was selected as 95% and P<0.05 was considered to indicate a statistically significant difference (23).

## Results

### Patient characteristics

In total, 40 adult patients with COPD, 9 female (22.5%) and 31 male (77.5%) with an average age of 65.0±14.50 years, volunteered for the study. The general features and plasma orexin-A and ghrelin levels of the patients are shown in Table I. Plasma orexin-A levels in male and female patients were found to be 1.3±0.37 and 1.4±0.13 ng/ml, respectively, whereas the average plasma ghrelin levels were 25.9±7.31 and 27.3±8.54 ng/ml, respectively. No statistically significant differences were observed between the two groups with regard to age, time course of COPD diagnosis and plasma orexin-A and ghrelin levels (P>0.05).

### Antropometric measurements

Anthropometric measurements of the patients are presented in Table II. When differences between genders were investigated, significant differences (P<0.05) were identified for the basal metabolic rate (P=0.004), lean body mass (P=0.004), total body water (P=0.004), skeletal-muscle weights (P=0.009)and intracellular (P=0.004) and extracellular (P=0.000) fluid amounts between the two groups.

### Orexin-A and ghrelin levels with BMI

**Figure 1:**
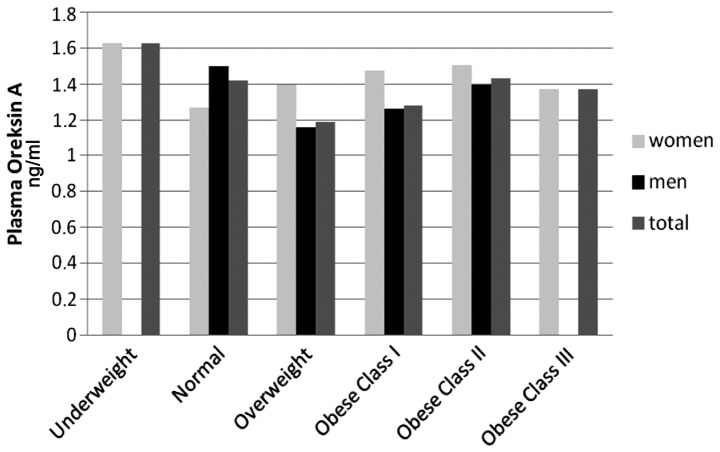
Distribution of plasma orexin-A levels according to patient BMI (P>0.05). BMI, body mass index.

**Figure 2:**
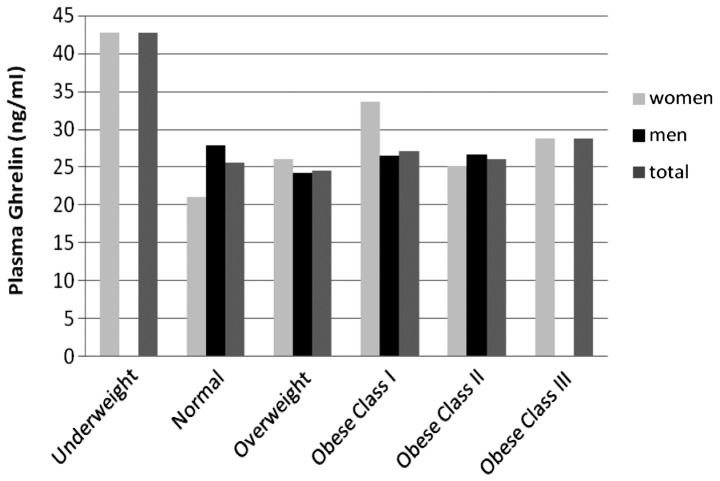
Distribution of plasma ghrelin levels according to patient BMI (P>0.05). BMI, body mass index.

The highest plasma orexin-A levels were identified in underweight female patients (BMI<18.5 kg/m^2^) and the lowest levels were identified in female patients with a normal BMI. However, as the number of underweight female patients was low, they were excluded from the statistical evaluation. Plasma orexin-A levels were only higher than those in female patients in male patients with a normal BMI (18.5–24.9 kg/m^2^). When the patients were classified according to their BMIs, the plasma orexin-A levels of the BMI groups were observed to be similar (P>0.05; Fig. 1). Similarly, no significant difference in ghrelin levels was identified according to the patient BMI classification (P>0.05; Fig. 2).

### Dietary intake and requirements

Daily average dietary energy and nutrient intakes and requirement meeting percentages are shown in Table III. Female patients met 89.6% of their daily energy requirement, whereas male patients met 79.0% of their energy requirement. While the rate of meeting the protein requirement was lower in female patients, no statistically significant difference was identified between the genders. The dietary saturated fatty acid intake was observed to be significantly higher in female patients than in male patients (P=0.05). The daily average dietary fibre intake was ~21.4±11.94 g. No significant differences were identified between the groups with regard to vitamins A, E, C and B complex intakes. The results indicated that female patients met only 59.8% of their folic acid requirement, while male patients met only 42.6% of their calcium requirement. The calcium intake of female patients was found to be significantly higher than the male patients (P=0.03).

**Figure 3:**
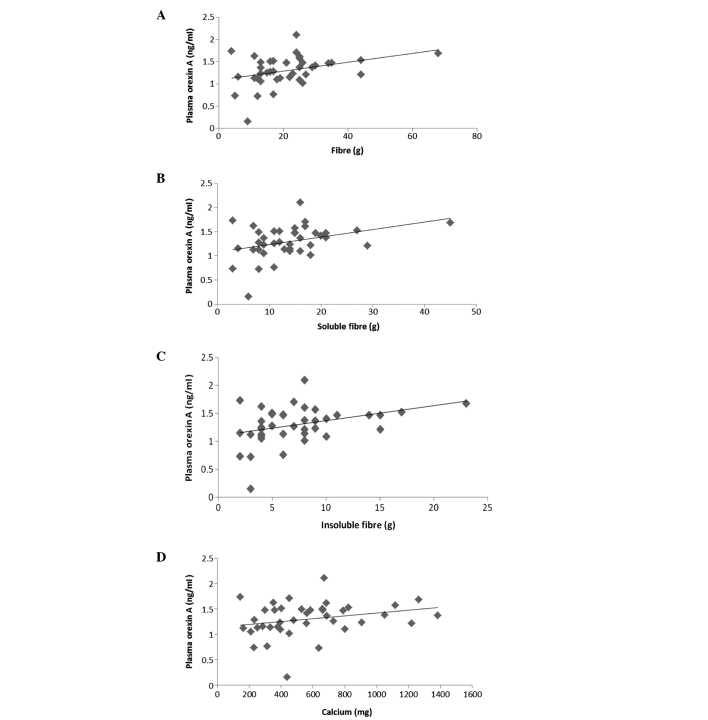
Correlation between plasma orexin-A levels and (A) daily dietary fibre intake (P=0.022, r=0.303), (B) dietary soluble fibre intake (P=0.029, r=0.033), (C) dietary insoluble fibre intake (P=0.024, r=0.335), and (D) dietary calcium intake (P=0.046, r=0.065).

Statistically significant differences were not observed between the plasma ghrelin levels and food intakes in the patients with COPD (P>0.05; Table IV). Correlations between plasma orexin-A levels and a number of dietary nutrient intakes are shown in Fig. 3. Plasma orexin-A levels were detected to be higher in patients with a higher dietary total fibre intake (r=0.303, P=0.022; Fig. 3A). Similarly, a higher dietary intake of soluble (r=0.033, P=0.029) and insoluble fibre (r=0.335, P=0.024) was found to be accompanied with higher plasma orexin-A levels (Fig. 3B and C). A similar correlation was observed between the daily calcium intake and plasma orexin-A levels (r=0.065, P=0.046; Fig. 3D; Table IV).

## Discussion

Malnutrition is a life-threatening health issue that is commonly observed in patients with COPD (3–8). Previous studies have demonstrated that the reason for these individuals being underweight is an imbalance between their energy expenditure and intake. Studies concerning the regulation of body weight emphasise the involvement of energy expenditure and appetite physiology, particularly neuropeptides that affect the nutritional status (24–26). The present study is of particular importance as, to the best of our knowledge, it is the first study investigating the association between plasma orexin-A and ghrelin levels, which are important polypeptides that stimulate food intake, with food consumption and body composition in patients with COPD in Turkey.

Previous studies on the association between plasma ghrelin levels with BMI and body composition in patients with COPD revealed contradictory results. Itoh *et al* (16) conducted a study to determine the plasma levels of ghrelin in patients with COPD and reported that plasma ghrelin levels of underweight patients with COPD were higher than those of normal weight individuals. In addition, a study conducted in China reported that total and active ghrelin levels in underweight patients with COPD were significantly higher when compared with normal weight COPD patients and the control group (27). In the present study, plasma ghrelin levels were highest in underweight female patients and lowest in female patients who had a normal BMI (P>0.05; Fig. 2). However, as the number of underweight female patients was not sufficient, they were not included in the statistical evaluation. In the study by Luo *et al* (28), plasma ghrelin levels were shown to positively correlate with the BMI and body fat percentage in patients with COPD and negatively correlate in control group patients. However, no significant correlation between the BMI and plasma ghrelin levels was observed in the present study (P>0.05; Fig. 2). Thus, further studies are required to fully elucidate the association between the plasma ghrelin levels and BMI in patients with COPD.

Matsuma *et al* (8) investigated plasma orexin-A levels in 20 patients with COPD and reported the plasma orexin-A levels of normal weight patients as 17.5±0.9 pg/ml and underweight patients as 14.1±0.5 pg/ml. Furthermore, the authors reported that plasma orexin-A levels positively correlated with BMI and body fat tissue (BMI, r=0.49, P=0.03; body fat tissue, r=0.53, P=0.02). These results indicated that plasma orexin-A levels may have an effect on the body composition of patients with COPD (8). However, in the present study, a statistically significant difference in plasma orexin-A levels with regard to BMI was not identified (P>0.05; Fig. 1). Although limited studies have focused on the association between plasma orexin-A levels and BMI in patients with COPD, studies on obese individuals have reported a negative correlation between BMI and plasma orexin-A levels (29,30).

The nutritional status of patients with COPD is an important factor that affects the onset of symptoms and prognosis of the disease. An increase in the oxygen demand in these patients also increases their energy requirement (31). However, the medication administered and short and frequent ventilation leads to insufficient food consumption (32). In a study conducted on 41 patients with COPD, individuals were found to have an insufficient energy intake (33). In addition, a study on 251 patients with COPD in Korea determined that the COPD patients met 66.76% of their daily energy requirement (31). In the present study, it was observed that patients with COPD met 81.3% of their daily energy requirement (Table III). Although malnutrition is commonly observed in COPD patients, it is positive that they meet the majority of their energy requirements. However, the protein, calcium and magnesium intakes, which may have an effect on muscle strength and respiratory functions, were found to be insufficient (Table III). Therefore, planning and implementation of nutritional therapy is of vital importance in COPD and requires dietician support.

Orexin contributes to respiratory control via increasing ventilation (32). Plasma orexin-A levels differ in COPD patients in the stable phase and in the situation of hypercapnic respiratory failure. While Matsumura *et al* (8) reported that patients with COPD in stable phase had lower plasma orexin-A levels compared with a control group, Zhu *et al* (34) reported that COPD patients with hypercapnic respiratory failure had higher plasma orexin levels compared with normal individuals.

Dietary nutrient intakes have been shown to affect the respiratory functions of patients with COPD (35,36). In particular, dietary fibre intake in adults is considered to have beneficial effects on chronic respiratory symptoms (37). However, sufficient information concerning an association between fibre consumption with pulmonary functions and COPD has not been demonstrated. In a previous study, individuals with the highest fibre consumption were reported to have higher forced expiratory volumes in one second compared with other individuals. Furthermore, a positive correlation has been demonstrated between the plasma levels of certain nutritional elements, including vitamin C, vitamin D, calcium, vitamin E, and respiratory coefficients (38). In a study conducted on 278 adults, the daily dietary intake of calcium and the risk of developing of COPD exhibited a negative correlation (34).

In the present study, the daily dietary intake of fibre and calcium, which positively affect pulmonary functions, and plasma orexin-A levels were demonstrated to be positively correlated. This correlation is hypothesised to have arisen from the effects of orexin-A, as well as fibre and calcium, on respiratory functions. As the number of studies on this subject is limited, the results of the present may provide useful information for future studies.

## Figures and Tables

**Table I:** General features and the plasma orexin-A and ghrelin levels of the patients.

	Female (n=9)		Male (n=31)		Total (n=40)		
				
Feature	Mean ± SD	Median/IQR	Mean ± SD	Median/IQR	Mean ± SD	Median/IQR	P-value
Age (years)	61.3±8.54	62.0±11.50	65.5±8.82	65.0±14.00	64.6±8.83	65.0±14.50	0.237
Time course of COPD diagnosis (months)	42.8±39.92	60.0±69.00	55.0±49.99	54.0±92.00	52.3±47.70	57.0±79.75	0.436
Orexin-A (ng/ml)	1.4±0.13	1.38±0.20	1.3±0.37	1.25±0.40	1.3±0.34	1.32±0.37	0.308
Ghrelin (ng/ml)	27.3±8.54	26.2±10.90	25.9±7.31	27.9±13.90	26.2±7.51	27.0±12.96	0.771

IQR, interquartile range; COPD, chronic obstructive pulmonary disease.

**Table II:** Body composition of the patients.

	Female (n=9)		Male (n=31)		Total (n=40)		
				
Body feature	Mean ± SD	Median/IQR	Mean ± SD	Median/IQR	Mean ± SD	Median/IQR	P-value
Body weight (kg)	72.2±19.45	68.1±28.00	78.7±17.02	76.4±19.60	77.2±17.55	74.5±21.60	0.271
Height (cm)	160.6±4.88	160.0±9.00	168.7±7.76	167.0±11.00	166.9±7.94	166.0±11.75	0.006^a^
BMI (kg/m^2^)	28.3±8.98	26.3±12.40	27.9±4.52	26.6±6.40	27.9±5.69	26.6±6.77	0.674
BMR (kcal)	1370.7±354.66	1295.0±154.30	1526.6±170.50	1545.0±218.00	1491.5±229.2	1488.0±272.00	0.004^a^
Waist/hip ratio	0.9±0.07	0.9±0.10	0.9±0.05	0.9±0.07	0.9±0.05	0.9±0.07	0.435
Fat mass (kg)	25.9±10.73	27.6±15.60	27.4±11.89	25.6±13.60	27.1±11.53	26.1±13.60	0.796
Fat (%)	35.6±10.57	37.6±20.00	31.9±6.33	31.1±10.10	32.8±7.49	31.4±10.90	0.271
Lean body mass (kg)	46.3±16.43	42.8±7.10	53.6±7.89	54.4±10.10	51.9±10.61	51.8±12.60	0.004^a^
Total body water (kg)	33.5±10.55	31.4±5.20	39.6±5.81	40.2±7.30	38.2±7.44	38.4±9.10	0.004^a^
Skeletal-muscle weight (kg)	26.8±13.37	22.9±4.60	29.5±4.66	30.1±5.40	28.9±7.16	28.8±7.40	0.009^a^
Intracellular fluid amount (l)	21.5±9.74	19.1±3.80	24.2±3.58	24.6±4.10	23.6±5.53	23.4±6.02	0.004^a^
Extracellular fluid amount (l)	12.0±1.10	12.3±1.90	15.4±2.25	15.6±2.80	14.6±2.49	14.7±3.78	0.000^a^

a Differences of anthropometric measurements between genders was compared and it was found statistically important (P<0.05).

IQR, interquartile range; BMI, body mass index; BMR, basal metabolic rate.

**Table III:** Daily dietary energy, nutrient intakes and requirement meeting percentages.

Energy and Nutrient	Female (n=9)			Male (n=31)			Total (n=40)			
		
	Mean ± SD	Median/IQR	RDA %	Mean ± SD	Median/IQR	RDA %	Mean ± SD	Median/IQR	RDA %	P-value
Energy (kcal)	1696.9±469.78	1559.8±798.00	89.6	1716.9±735.89	1606.5±652.00	79	1712.5±679.64	1593.1±639.00	81.3	0.686
Total protein (g)	59.1±20.43	53.1±34.00	57.8	60.9±26.92	55.9±37.00	62.9	60.5±25.37	55.6±36.00	61.7	0.859
Total lipid^b^ (g)	83.1±30.68	67.8±50.00	131–157	69.2±33.11	60.1±39.00	97–116	72.4±32.72	65.0±36.00	104–125	0.159
SFA^b^ (g)	31.2±12.94	28.9±20.00	185–212	22.2±10.35	21.2±17.00	115–132	24.3±11.45	21.9±16.00	131–150	0.05^a^
MUFA^b^ (g)	30.9±15.34	22.4±28.00	97–121	25.3±12.63	22.5±20.00	70–87	26.5±13.29	22.5±20.00	76–95	0.308
PUFA^b^ (g)	15.8±7.53	13.5±15.00	94–107	16.8±13.79	14.4±9.00	87–99	16.6±12.58	14.0±10.00	88–101	0.974
Ω3 (g)	1.7±1.01	1.74±1.00	153.0	1.32±0.99	1.06±1.00	82.0	1.4±0.99	1.1±1.00	97.9	0.206
Ω6 (g)	13.7±6.72	11.7±12.00	124.7	15.5±13.24	13.3±9.00	110.6	15.1±12.02	12.9±9.00	113.8	0.734
Cholesterol^b^ (mg)	204.4±89.39	188.4±170.00	<300	259.0±373.47	148.2±210.00	<300	246.7±330.86	170.4±199.00	<300	0.466
Fibre (g)	20.5±7.38	20.6±15.00	95.2	21.6±13.06	19.1±12.00	71.7	21.4±11.94	19.8±13.00	77.0	0.783
Soluble fibre (g)	6.3±2.13	5.9±4.00		7.5±4.89	6.4±5.00		7.2±4.42	6.36±5.00		0.961
Insoluble fibre (g)	14.0±5.37	14.6±11.00		14.0±8.42	13.9±9.00		14.0±7.77	6.4±9.00		0.571
Vitamin A (μg)	1172.9±768.70	819.6±1487.00	167.6	1515.4±3858.05	645.4±1020.00	168.4	1438.4±3404.68	700.8±1041.00	168.2	0.190
Vitamin E (mg)	13.86±6.95	11.0±11.00	87.6	16.8±14.79	14.0±10.00	111.8	16.9±13.88	14.0±10.00	106.4	0.476
Vitamin B1 (mg)	0.8±0.29	0.6±0.60	69.5	0.9±0.57	0.8±0.49	75.1	0.8±0.52	0.8±0.51	73.9	0.686
Vitamin B2 (mg)	1.3±0.62	1.1±1.30	118.5	1.2±0.84	1.0±0.74	91.6	1.2±0.79	1.0±0.74	97.7	0.446
Vitamin B6 (mg)	1.4±0.62	1.2±0.88	93.3	1.3±0.69	1.2±0.63	78.7	1.3±0.67	1.2±0.70	81.9	0.859
Vitamin B12 (mg)	3.8±2.73	2.6±4.80	157.1	5.6±14.33	2.8±3.08	234.7	5.2±12.65	2.8±3.28	195.4	0.639
Niacin (mg)	13.3±7.98	12.5±14.00	94.8	11.5±7.23	10.4±11.00	72.0	11.9±7.33	10.4±11.00	91.2	0.560
Folic acid (μg)	261.9±89.61	253.9±137.00	59.8	314.1±151.2	265.6±144.00	80.2	302.3±140.43	265.5±134.00	83.5	0.339
Vitamin C (mg)	164.9±130.74	107.8±237.00	219.9	144.6±184.72	83.1±148.00	160.6	149.1±172.71	85.9±191.00	173.9	0.447
Calcium (mg)	798.3±366.52	687.5±675.00	68.5	507.2±267.61	451.5±370.00	42.6	572.7±312.72	506.3±382.0	48.4	0.03^a^
Magnesium (mg)	265.1±137.76	196.7±196.00	82.8	244.3±186.88	212.5±113.00	58.2	248.9±175.59	212.5±138.0	63.7	0.507
Phosphorus (mg)	1125.5±459.63	869.3±795.0	160.8	977.3±473.69	938.4±543.0	139.6	1010.6±468.89	936.8±612.0	144	0.466

a P<0.05.

b Dietary Guidelines for Turkey (20).

IQR, interquartile range; RDA, recommended daily allowance; SFA, saturated fatty acids; MUFA, monounsaturated fatty acids; PUFA, polyunsaturated fatty acids.

**Table IV:** Regression models between plasma orexin-A levels and the daily dietary intake of nutrients.

Plasma orexin-A associated parameters	Constant	Regression Coefficient	P-value
Fibre	1.082	0.0100	0.022
Soluble fibre	1.081	0.0150	0.029
Insoluble fibre	1.104	0.0268	0.024
Calcium	1.140	0.0003	0.046

When the statistical analyses were examined, simple linear regression models were created to estimate the plasma orexin-A values from the values of the associated parameters. The models shown in Table IV were found to be statistically significant (P<0.05).
